# The Association of Physical Activity to Cognitive Performance in African American Older Adults

**DOI:** 10.1093/geroni/igaf122.4024

**Published:** 2025-12-31

**Authors:** Rio Tate, Brent Small, William Haley

**Affiliations:** University of South Florida, Tampa, Florida, United States; University of South Florida, Tampa, Florida, United States; University of North Carolina at Chapel Hill, Chapel Hill, North Carolina, United States

## Abstract

The number of older adults in the United States is expected to increase in the years to come. As we age, cognitive performance is noted to decline. African American older adults are more likely than non-Hispanic Whites to experience cognitive impairment and dementia. It is known that physical activity is a modifiable risk factor for dementia. It is not known the extent to which physical activity engagement is related to cognitive performance in African American older adults, and which variables, if any, moderate this association. To answer this question, the authors examined the association between three intensities of physical activity (mild, moderate, and vigorous) and four domains of cognitive performance (episodic memory, working memory, total mental status, and total cognition). Data were analyzed at five time points using ten years of data from the Health and Retirement Study. Data was also analyzed at the between-person and within-person level. Results indicated that greater engagement in physical activity was associated with greater cognitive performance across a variety of domains. Younger age, higher years of education, and female gender moderated this association. Implications from this study indicate the effectiveness of engaging in a modifiable risk factor of dementia with regards to higher cognitive performance in minority older adults. Downstream cross-racial comparisons examining the effectiveness of physical activity on cognitive performance are therefore based in more comprehensive data analysis.

